# Correction: TiO_2_ nanotube stimulate chondrogenic differentiation of limb mesenchymal cells by modulating focal activity

**DOI:** 10.1038/s12276-019-0364-6

**Published:** 2020-01-20

**Authors:** Dongkyun Kim, Bohm Choi, Jinsoo Song, Sunhyo Kim, Seunghan Oh, Eun-Heui Jin, Shin-Sung Kang, Eun-Jung Jin

**Affiliations:** 1 Department of Biological Sciences, College of Natural Sciences, Wonkwang University, Iksan, 570-749 Korea; 2 Department of Prosthodontic Dentistry, Uijeongbu St. Mary’s Hospital, The Catholic University of Korea, Uijeongbu, 480-717 Korea; 3 Department of Dental Biomaterials, College of Dentistry, Wonkwang University, Iksan, 570-749 Korea; 4 School of Biological Sciences, University of Ulsan, Ulsan, 680-749 Korea; 5 Daegu Center, Korea Basic Science Institute, Daegu, 702-701 Korea

**Keywords:** Cell signalling, Cell growth

**Correction to: Experimental & Molecular Medicine**

10.3858/emm.2011.43.8.051, published online 16 June 2011

After online publication of this article, the authors noticed an error in the Fig. 2B. We accidently added the unnecessary GAPDH band in Fig. 2b.

**Figure 1:**
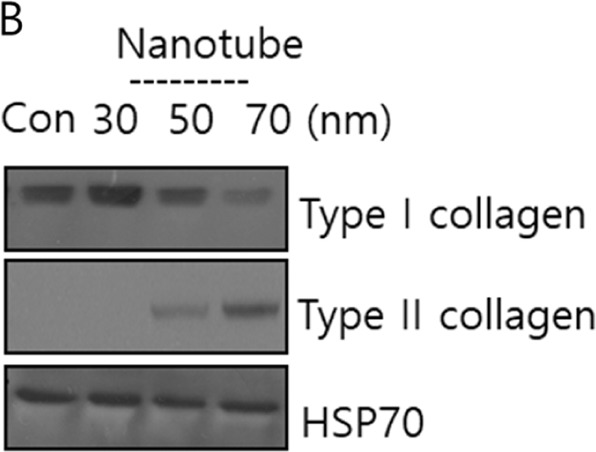
Fig. 2

The authors apologize for any inconvenience caused.

